# Outcomes for continuous subcutaneous insulin infusion users in young adults from lower socioeconomic backgrounds

**DOI:** 10.1002/edm2.252

**Published:** 2021-05-14

**Authors:** Alexis M. McKee, Stewart G. Albert, Noor Al‐Hammadi, Leslie J. Hinyard

**Affiliations:** ^1^ Division of Endocrinology Metabolism & Lipid Research Washington University School of Medicine in St. Louis St. Louis MO USA; ^2^ Division of Endocrinology Diabetes & Metabolism Saint Louis University School of Medicine St. Louis MO USA; ^3^ Department of Health & Clinical Outcomes Research Saint Louis University School of Medicine St. Louis MO USA; ^4^ Advanced HEAlth Data (AHEAD) Institute Saint Louis University St. Louis MO USA

**Keywords:** continuous subcutaneous insulin infusion, insulin pump, medicaid, socioeconomic status, type 1 diabetes

## Abstract

**Objective:**

Diabetes technology is available and its efficacy and safety have been demonstrated; however, there is little evidence as to how this technology is being utilized and its effectiveness in vulnerable populations. This study evaluated differences in outcomes for young adults in the United States (U.S.) from lower socioeconomic (SES) backgrounds with type 1 diabetes (T1D) managed on continuous subcutaneous insulin infusion (CSII) versus multiple daily injections (MDI) or fixed‐dose insulin (FDI).

**Research design, methods and participants:**

Utilizing the Optum® de‐identified Electronic Health Record data set between 2008 and 2018 to perform a retrospective, cohort study, we identified 805 subjects with T1D aged 18–30 years with Medicaid. We evaluated median difference in HbA1c between CSII and MDI/FDI users for 24 months. Predictors of diabetic ketoacidosis (DKA)‐associated hospitalizations by CSII use were evaluated using logistic regression.

**Results:**

CSII users showed statistically significant lower median HbA1c values at 24 months of follow‐up compared to individuals on MDI/FDI. Non‐white individuals were at lower odds of receiving treatment with CSII. Subjects on CSII were not more likely to be hospitalized for DKA compared to subjects treated with MDI/FDI. Older subjects were at lower odds of being hospitalized for DKA. Males and subjects followed by Endocrinologists were at higher odds of being hospitalized for DKA.

**Conclusions:**

Young adults with T1D from lower SES backgrounds show improved glycaemic control when in CSII compared to MDI/FDI without increases in hospitalizations for DKA.

## Graphical abstract

Young adults with T1D from lower SES backgrounds show improved glycaemic control when on CSII compared to MDI/FDI without increases in hospitalizations for DKA.

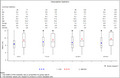

## 1 INTRODUCTION

The treatment of type 1 diabetes (T1D) is rapidly evolving with the development of novel technologies such as continuous subcutaneous insulin infusion (CSII), continuous glucose monitoring (CGM), sensor augmented pumps (SAP) and hybrid closed‐loop (HCL) systems.^1^ Despite these exciting advances, adoption of these technologies and access to them has not permeated to all segments of the population.^2^, ^3^ Large database and registry data have demonstrated that many youth and young adults with T1D do not meet established haemoglobin A1c (HbA1c) goals.^4^, ^5^ In fact, according to data from the T1D Exchange Registry, between 2016 and 2018, only 17% of youth achieved the American Diabetes Association (ADA) HbA1c goal <7.5%.^6^ While reasons for poor glycaemic control in this group are multifactorial, lack of diabetes technology utilization may play a role.

Diabetes technologies as stand‐alone insulin pumps and CGMs in addition to systems in which the pump and sensor fully communicate are being increasingly utilized to treat T1D. After almost four decades since insulin pumps became commercially available starting with Medtronic's Minimed 502 in 1983, there are substantial data that individuals with T1D on CSII demonstrate better HbA1c outcomes compared to MDI.^7^ Furthermore, subjects with T1D on CSII demonstrate improvements in microvascular outcomes including retinopathy and peripheral neuropathy compared to management with multiple daily injections (MDI).^8^ CGM was next to make its way to the T1D community and has demonstrated numerous benefits including HbA1c reduction, reduction of glycaemic variability and less hypoglycaemia.^9^, ^10^ Moreover, individuals with CGM who are switched from MDI to CSII spend more time in range (TIR) defined as the glucose concentration 70–180 mg/dL compared to those on CGM and MDI.^11^ Results seem to improve further the more automated the system, as seen in SAP therapy which has been shown to reduce HbA1c and time spent in hypoglycaemia.^12^ Most recently, HCL systems are available and illustrate encouraging time in range data.^13^, ^14^

While CSII, CGM, SAP and HCL systems have demonstrated real benefit in T1D patients, there are no uniform guidelines or treatment algorithms within the United States to guide practitioners regarding how to best deploy these technologies to the patients who would benefit most. According to the American Diabetes Association (ADA) 2021 guidelines, insulin pump therapy may be considered for all adults and youth with T1D who can safely manage the device.^15^ This guidance is inconsistent with older Endocrine Society 2016 and American Association of Clinical Endocrinologists (AACE) 2018 guidelines which included more restrictions around the definition of an ideal pump candidate.^16^, ^17^ Even in countries with nationalized healthcare systems and guidance on who qualifies for pump therapy, there is low utilization of such devices. For instance, the National Institute for Health and Clinical Excellence (NICE) published guidelines for CSII use in 2008 in the United Kingdom (NICE TA151, UK Best Practice Guide). Despite these recommendations, an audit in 2011 revealed an estimated prevalence of 6% for CSII utilization.^18^

While diabetes technology is available and the data regarding its efficacy and safety have also been demonstrated, what has not been extensively shown is what adult cohort of the T1D community is accessing these treatment modalities and what influences their availability especially to the most vulnerable groups of the T1D population. It does not appear that there is much diversity in the technology trials to date and some studies do not even list baseline demographics.^13^, ^14^ Additionally, it appears that there are clear racial and ethnic biases regarding who receives newer technologies even when controlling for SES.^3^ The data regarding racial and ethnic biases are predominantly from the paediatric literature. In a study by Willi et al using data from the T1D Exchange Clinic Network, fewer Black compared to white children across all income strata were on insulin pumps. Higher HbA1c values were seen even in high‐income Black families, perhaps because fewer of them were managed with insulin pump therapy. Black children with private insurance were less likely to be on insulin pumps compared with white children without private insurance.^3^ In another study in a paediatric population of subjects with T1D by Lin et al, subjects of non‐Hispanic white race and higher socioeconomic status were more likely to be placed on pumps in the first year following diagnosis.^19^ In a recent study examining racial‐ethnic inequity in young adults with T1D, Black young adults had the lowest insulin pump use, despite similar rates of public insurance as Hispanic young adults.^20^

When diabetes technology companies reach out to insurance companies through their managed care teams, they lobby for coverage of devices by showing clinical outcome data. Medicaid is health care in the United States funded by individual states and the federal government to eligible low‐income children, adults, pregnant women and people with disabilities. There is variable coverage of diabetes technology for individuals with T1D depending on the state in which they reside and according to individual Medicaid plan. In order to expand insurance coverage for diabetes technology and provide more emphasis for why racial‐ethnic biases in prescribing patterns of diabetes technology need further investigation, data on clinical outcomes in a lower socioeconomic young adult patient population with T1D need to be shown.

This study evaluated whether young adults on Medicaid with T1D had better clinical outcomes as defined by HbA1c and hospitalizations for diabetic ketoacidosis (DKA) when on CSII compared to subjects on MDI or fixed‐dose insulin (FDI).

## 2 RESEARCH DESIGN AND METHODS

### 2.1 Data source

We performed a retrospective cohort study using the Optum® de‐identified Electronic Health Record (EHR) data set.^21^ The data set contained EHR data from 5 million adults (age 18 and older), nationally distributed across the United States. EHR data contains ICD‐9 and ICD‐10 codes, prescription medication orders, vital signs, laboratory results, procedure codes and demographic measures. The Optum® de‐identified EHR data set pulls electronic medical records from integrated delivery networks and facilities spanning the United States and processes and standardizes field for use in research. The data are longitudinal and have been used in hundreds of publications in multiple disease areas including diabetes.^22^

### 2.2 Study sample

The study sample was limited to T1D patients with an encounter between 2008 and 2018 and had activity for at least two years after first encounter date. Subjects aged 18 to 30 years, on Medicaid, and with a diagnosis of T1D were identified. Medicaid was used as a proxy for lower SES.^23^ Subjects with a diagnosis code of pregnancy and those who received glucose‐lowering medications for type 2 diabetes (T2) were excluded from the analysis.

### 2.3 Measures

Prescription and diagnosis codes for all variables are listed under supplemental material.

### 2.4 Exposure

Treatment modality was classified as CSII versus MDI or FDI. Subjects were assigned to CSII or MDI or FDI based on encounter diagnosis codes (see Table S1 for a list of diagnosis codes). For subjects with a diagnosis code for CSII, the first record indicating CSII was considered the date of insulin pump initiation. Patients on MDI and FDI were combined as the non‐CSII group and were defined using prescription codes, and the date of enrolment in the cohort was considered their treatment start date (see Table S2). All laboratory results and hospitalization data prior to this treatment start date were excluded from this analysis.

### 2.5 Outcomes

The primary outcome was HbA1c (%) measurements obtained at 3–6, 7–12, 13–18 and 19–24 months following the assignment of treatment modality. HbA1c values were considered if occurred at least 3 months after CSII prescription. We used the last HbA1c for every patient with multiple HbA1c records per each 3‐month interval. For graphical illustrations, laboratory data in the form of HbA1c were analysed at 6‐month intervals.

The secondary outcome was hospitalization for DKA. Unique DKA encounters were defined as an inpatient diagnosis code for DKA corresponding to a distinct inpatient visit encounter ID. We calculated the total number of unique DKA episodes of DKA per subject over the 24‐month follow‐up period.

### 2.6 Covariates

We controlled for the age, race, ethnicity, gender and income of the subject. Age was classified as 18–26 years and 27 or older at the time of first encounter. This was based on knowledge that parental healthcare coverage for dependent children ends after age 26. Blacks, Asians and other races were grouped into one racial category and compared to whites in this analysis. Ethnicity was also re‐categorized into Hispanics vs. other ethnicities. Subjects making less than $45,000 a year were considered low‐income individuals. Because adult Endocrinologists may differ in their approach to prescribing CSII to their patients when compared to physicians from other specialties, a composite variable was generated specifying if the subject had at least one encounter with an adult Endocrinologist and was adjusted for in the analysis.

### 2.7 Statistical analyses

Characteristics and outcomes of the study sample overall and stratified by CSII use are presented as medians and interquartile ranges or frequencies and percentages, as appropriate. Bivariate comparisons for all variables by CSII use were examined using the Mann‐Whitney U tests for continuous variables and Chi‐square tests for categorical variables. Differences in HbA1c between CSII and MDI/FDI users were compared at baseline and at each determined interval for the 24‐month follow‐up period. Univariate and multivariable logistic regression analyses were used to identify factors associated with CSII prescription and DKA‐related hospital admissions. A histogram was used to illustrate the HbA1c at 6‐month intervals. Model fitness was tested using the likelihood test. All tests were two‐sided, and the alpha level of significance was set at 0.05. All analyses were done using SAS 9.4 (SAS Institute Inc., Cary, NC, USA).

## 3 RESULTS

Demographic characteristics of the 805 subjects that met inclusion criteria are shown in Table 1. Overall, 65.8% were between age 18 and 26 years, 54.9% were female, 64.6% were white, 8.5% were Hispanics and 77% reported annual household income less than $45,000. Of the cohort, 45.1% were treated by adult Endocrinologists at least once. Subjects managed by CSII accounted for 13% of the cohort. Subject characteristics by treatment modality at first encounter are described in Table 1. When examining the distribution of subjects across the two treatment arms, there were differences in treatment modality by race and specialty follow‐up. CSII users were more likely to be white (84.8%) and followed by an adult Endocrinologist (66.7%). The number of enrollees by year along with the number of subjects with insulin pump initiation by year within the cohort is shown in Table S3.

**TABLE 1:** Demographic Characteristics (N = 805)

Baseline Characteristics	All	CSII	MDI/Fixed	P‐value
	N, %	N, % (105, 13.0)	N, % (700, 87.0)	
Age (in years)				
18–26	530 (65.8)	78 (74.3)	452 (64.6)	**0.050**
27–30	275 (34.2)	27 (25.7)	248 (35.4)	
Gender				
Female	442 (54.9)	63 (60.0)	379 (54.1)	0.261
Male	363 (45.1)	42 (40.0)	321 (45.9)	
Race				
White	520 (64.6)	89 (84.8)	431 (61.6)	**<.0001**
Other Races	285 (35.4)	16 (15.2)	269 (38.4)	
Ethnicity				
Hispanic	68 (8.5)	4 (3.8)	64 (9.1)	0.067
Other Ethnicities	737 (91.5)	101 (96.2)	636 (90.9)	
Income				
$45,000+	185 (23.0)	20 (19.1)	165 (23.6)	0.304
<$45,000	620 (77.0)	85 (80.9)	535 (76.4)	
Specialty				
Endocrinology	363 (45.1)	70 (66.7)	293 (41.9)	**<.0001**
Other Specialties	442 (54.9)	35 (33.3)	407 (58.1)	

Note

Chi‐Square test.

Values in bold font indicate significance at 0.05.

**FIGURE 1:**
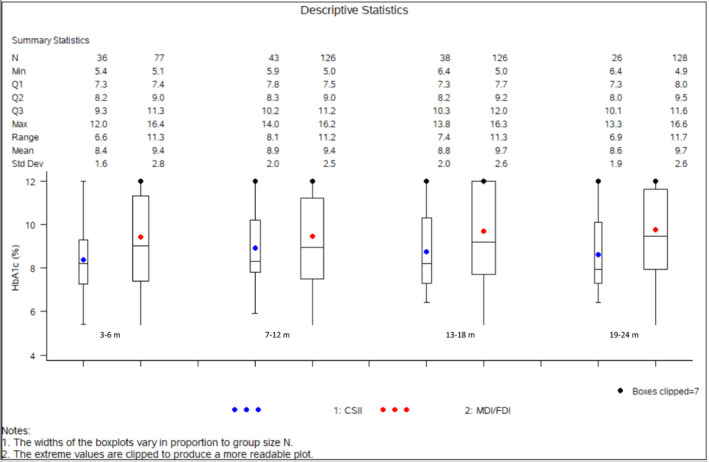
Mean HbA1c (± SD) stratified by time period and treatment modality. HbA1c: haemoglobin A1c, CSII: continuous subcutaneous infusion, blue. MDI/FDI: multiple daily injections/fixed‐dose insulin, red

The median HbA1c in CSII users was 8.0% (IQR: 7.3–10.1) versus 9.5% in the MDI/FDI group (IQR: 8.0–11.6) (*P *= 0.021) at 19–24 months of follow‐up. Comparison of the median HbA1c levels by treatment modality at each follow‐up point is illustrated in Table 2, Figure 1.

**TABLE 2:** Median HbA1c (%) (within 24 months of follow‐up) by treatment modality

Month	CSII		N	MDI/FDI	*P*‐value
	N	Median (IQR)		Median (IQR)	
3 to 6	36	8.2 (7.3–9.3)	77	9.0 (7.4–11.3)	0.065
7 to 12	43	8.3 (7.8–10.2)	126	9.0 (7.5–11.2)	0.265
13 to 18	38	8.2 (7.3–10.3)	126	9.2 (7.7–12.0)	**0.041**
19 to 24	26	8.0 (7.3–10.1)	128	9.5 (8.0–11.6)	**0.021**

Note

Mann‐Whitney U test.

Values in bold font indicate significance at 0.05.

Table 3 presents information on the number of DKA‐associated hospitalizations. No significant differences were found in DKA admissions between the CSII users and the MDI/FDI group during the 24‐month follow‐up period (31.4% vs 25.1%, *P *= 0.171 respectively).

**TABLE 3:** Events of DKA (within 24 months of follow‐up) by treatment modality

Number of DKA Admissions	CSII	MDI/Fixed	*P* value
	N, %	N, %	
0	72 (68.6)	524 (74.9)	
1	14 (13.3)	79 (11.3)	
2	7 (6.7)	28 (4.0)	
3	3 (1.9)	15 (2.1)	
4	2 (1.9)	12 (1.7)	
5	0 (0.0)	9 (1.3)	
≥6	7 (6.7)	33 (4.7)	
Hospitalization for DKA			
Yes	33 (31.4)	176 (25.1)	0.171
No	72 (68.6)	524 (74.9)	

Determinants of receiving CSII as a treatment modality are presented in Table 4. Subjects followed by adult Endocrinologists were more likely to receive insulin pump treatment (aOR = 2.67, 95% CI: 1.71, 4.15). Non‐white subjects were at lower odds of receiving CSII (aOR = 0.30, 95% CI: 1.17, 0.52).

**TABLE 4:** Predictors of CSII Use (Logistic Regression).

	cOR, 95%CI (LL, UL)	aOR, 95%CI (LL, UL)
Other Races (reference = White)	**0.29 (0.17, 0.50)**	**0.30 (0.17, 0.52)**
Hispanic (reference = Non‐Hispanic / others)	0.39 (0.14, 1.10)	0.44 (0.15, 1.26)
27+ years (reference = 18–26 years)	0.63 (0.40, 1.00)	0.66 (0.41, 1.06)
Male (reference = Female)	0.79 (0.52, 1.20)	0.80 (0.52, 1.24)
Low income (<$45,000) (reference = $45,000+)	1.31 (0.78, 2.20)	1.53 (0.89, 2.61)
Endocrinologist (reference = Other Specialties)	**2.78 (1.80, 4.28)**	**2.67 (1.71, 4.15)**

Note

Values in bold font indicate statistical significance.

Predictors of hospitalization for DKA within the 24‐month follow‐up period are shown in Table 5. Males were at higher odds of hospitalization for DKA (aOR = 1.57, 95% CI: 1.14, 2.18). The odds of admission for DKA were higher among subjects seen by an adult Endocrinologist in this cohort (aOR = 1.76, 95% CI: 1.27, 2.44). Older subjects were at lower odds of DKA (aOR = 0.57, 95% CI: 0.40, 0.82).

**TABLE 5:** Predictors of Hospitalizations for DKA within 24 months of follow‐up (Logistic Regression).

	cOR, 95%CI (LL, UL)	aOR, 95%CI (LL, UL)
CSII Use (reference = MDI/FDI)	1.37 (0.87, 2.13)	1.13 (0.71, 1.81)
Other Races (reference = White)	1.00 (0.72, 1.39)	1.03 (0.73, 1.45)
Hispanic (reference = Non‐Hispanic / others)	0.65 (0.35, 1.22)	0.63 (0.33, 1.20)
27+ years (reference = 18–26 years)	**0.59 (0.42, 0.84)**	**0.57 (0.40, 0.82)**
Male (reference = Female)	**1.51 (1.10, 2.07)**	**1.57 (1.14, 2.18)**
Low income (<$45,000) (reference = $45,000+)	1.47 (0.99, 2.19)	1.50 (0.99, 2.26)
Endocrinologist (reference = Other Specialties)	**1.81 (1.32, 2.49)**	**1.76 (1.27, 2.44)**

Note

Values in bold font indicate statistical significance.

## 4 DISCUSSION

In our sample of young adults with T1D on Medicaid, we found that median HbA1c levels were statistically significantly lower in individuals managed with CSII compared to MDI/FDI at 24 months of follow‐up. These results are consistent with an analysis of randomized clinical trials comparing CSII with MDI in subjects with T1D in which CSII was shown to lead to statistically lower HbA1c values regardless of whether regular human insulin or rapid‐acting analogue insulin was utilized.^7^ In addition to being statistically significant, the difference between median HbA1c values in the CSII group versus the MDI/FDI group is clinically relevant. According to landmark data from the Diabetes Complications and Control Trial (DCCT) and the Epidemiology of Diabetes Interventions and Complications (EDIC) follow‐up, intensive glucose control reduces the risk of microvascular complications.^24^ In fact, the risk reduction is non‐linear, and thus, even greater risk reduction is seen in those individuals with initial higher HbA1c levels. This is particularly meaningful in the lower SES population that we describe, who if faced with further disabilities due to microvascular changes including blindness, dialysis and amputation, the further down on the socioeconomic ladder he or she will fall. Ultimately, this will create an economic burden and result in increased healthcare utilization.

Risk for DKA was not greater in the CSII group; a significant finding given the reduced utilization of CSII in the Non‐white population. It is possible that there is inherent bias by practitioners to avoid prescribing CSII to Non‐white individuals and low SES groups due to a fear that those populations may not be able to successfully operate the technology. The lack of increased risk of DKA for persons on CSII may provide more assurance to providers that they can be prescribed to patients for whom technology has been historically withheld out of fear of adverse outcomes. Predictors of DKA included following with an adult Endocrinologist which may be explained by the fact that typically more complex individuals with T1D follow with specialists. Male subjects were also at increased odds for hospitalization for DKA which warrants further explanation. Older individuals were at less risk of DKA perhaps owing to more experience with CSII. Neither race nor ethnicity was a predictor for hospitalization for DKA. These findings are in contrast to systematic review examining predictors of DKA in adults with T1D which showed prevalence of DKA decreased with increasing age but was higher in non‐white ethnicities.^25^

Even though CSII has been available for several decades now, there appears to be low technology utilization among young adults with T1D and lower SES. Additionally, the majority of individuals with T1D in this cohort are followed by non‐Endocrinologists, perhaps due to the shortage of clinical Endocrinologists and/or due to the lack of commercial insurance on the part of the subjects. It is possible that low technology utilization is influenced by the lack of encounters or access to specialist care.

CSII does not appear to be prescribed at similar rates to Non‐white young adults with T1D from lower socioeconomic backgrounds managed by Medicaid. The reason for this warrants further investigation. This finding is in keeping with data from the T1D Exchange network which demonstrated large racial‐ethnic inequity in young adults with T1D, especially in Black participants. Compared to White participants, fewer Black and Hispanic individuals utilized CSII, with Black young adults having the lowest rates and highest HbA1c levels.^20^ T1D subjects on Medicaid followed by adult Endocrinologists were more likely to receive CSII.

This study has limitations. Although it is recognized that in the real world, individuals with T1D may alternate between therapies; treatment category was determined according the type of diabetes therapy at baseline entry in the study. The duration of 24‐month follow‐up does not account for crossovers between therapies over time. However, using baseline treatment assignment is likely to bias effect sizes towards the null. The results are also limited by the sample size and the number of available HbA1c values at each reference point. If a larger sample was available, it would be interesting to examine the disparities in patient characteristics in each treatment group in reference to HbA1c.

Another limitation of the study is that as investigators, we cannot account for the ever‐changing differences in CSII coverage by state and type of Medicaid insurance plan. CSII coverage changes frequently over time, by state and by individual plan.

The healthcare implications of this study are important and timely in an era of unprecedented advances in diabetes technology and a time of much discussion regarding social inequity in the United States. Further examination and study of prescribing biases and insurance boundaries to diabetes technology is needed. To make informed decisions about diabetes technology in adults with T1D, clinical trials that are more inclusive of minorities and those of lower SES are needed.

## 5 CONCLUSION

Statistically significant and clinically relevant lower median HbA1c values are seen in individuals on CSII versus MDI/FDI in this cohort of subjects with T1D on Medicaid. Further research into disparities in diabetes technology prescribing and predictors of successful CSII utilization in young adults with T1D from disadvantaged backgrounds is needed. Clinical trials involving diabetes technology should be more inclusive of young adults with T1D of lower SES.

## CONFLICT OF INTEREST

The authors have no conflicts of interest relevant to this article to disclose.

## AUTHOR CONTRIBUTION

A.M. and S.A. wrote the research proposal. N.A. conducted the data analyses. L.H. edited the manuscript. A.M. and N.A. are the guarantors of the work, as such, had full access to the data in the study and take responsibility for the integrity of the data and accuracy of the analysis.

## Supporting information

Table S1‐S3

## Data Availability

The data that support the findings of this study are available from Optum® de‐identified Electronic Health Record data set. Restrictions apply to the availability of these data, which were used under licence for this study. Data are available from the corresponding author with the permission of Optum® de‐identified Electronic Health Record data set.
