# Supplementary material for: Outcomes for continuous subcutaneous insulin infusion users in young adults from lower socioeconomic backgrounds
Source: Endocrinol Diabetes Metab. 2021 May 14;4(3):e00252. doi: 10.1002/edm2.252 (PMC8279595; doi:10.1002/edm2.252)
Supplement: Supplementary file 1 — Table S1‐S3 [file EDM2-4-e00252-s001.docx]

**SUPPLEMENTAL TABLES**

Supplemental Table 1: Encounter Diagnosis Codes

| **Category** | **Diagnosis Code** |
| --- | --- |
| T1D | ICD9 250.01, 250.03, 250.11, 250.13, 250.21, 250.23, 250.31, 250.33, 250.41, 250.43, 250.51, 250.53, 250.61, 250.63, 250.71, 250.73, 250.81, 250.83, 250.91, 250.93 |
|  | ICD10 E10.1, 10.10, 10.11, 10.2, 10.21, 10.22, 10.29, 10.3, 10.31, 10.311, 10.319, 10.32, 10.321, 10.3211, 10.3212, 10.3213, 10.3219, 10.329, 10.3291, 10.3292, 10.3293, 10.3299, E10.33, 10.331, 10.3311, 10.3312, 10.3313, 10.3319, 10.339, 10.3391, 10.3392, 10.3393, 10.3399, 10.34, 10.341, 10.3411, 10.3412, 10.3413, 10.3419, 10.349, 10.3491, 10.3492, 10.3493, 10.3499, 10.35, 10.351, 10.3511, 10.3512, 10.3513, 10.3519, 10.352, 10.3521, 10.3522, 10.3523, 10.3529, 10.353, 10.3531, 10.3532, 10.3533, 10.3539, 10.354, 10.3541, 10.3542, 10.3543, 10.3549, 10.355, 10.3551, 10.3552, 10.3553, 10.3559, 10.359, 10.3591, 10.3592, 10.3593, 10.3599, 10.36, 10.37, 10.37X1, 10.37X2, 10.37X3, 10.37X9, 10.39, 10.4, 10.40, 10.41, 10.42, 10.43, 10.44, 10.49, 10.5, 10.51, 10.52, 10.59, 10.6, 10.61, 10.610, 10.618, 10.62, 10.620, 10.621, 10.622, 10.628, 10.63, 10.630, 10.638, 10.64, 10.641, 10.649, 10.65, 10.69, 10.8, 10.9 |
| Pregnancy | ICD9 V22.0, 22.1, 22.2, 23.0, 23.1, 23.2, 23.3, 23.4, 23.41, 23.42, 23.49, 23.5, 23.7, 23.8, 23.81, 23.82, 23.83, 23.84, 23.85, 23.86, 23.87, 23.89, 23.9 |
|  | ICD10 Z33.1, 33.2, 33.3, 34.00, 34.01, 34.02, 34.03, 34.80, 34.81, 34.82, 34.83, 34.90, 34.91, 34.92, 34.93, 36, 36.0, 36.1, 36.2, 36.3, 36.4, 36.5, 36.81, 36.82, 36.83, 36.84, 36.85, 36.86, 36.87, 36.88, 36.89, 36.8A, 36.9 |
| Diabetic Ketoacidosis | ICD9 250.11, 250.12, 250.13 |
|  | ICD10 E10.10, 10.11 |

Supplemental Table 2: Grouping of Insulins and CSII

| **Category** | **Search Terms / Diagnosis Code** |
| --- | --- |
| Classes of Glucose-Lowering Medications for T2D | Sulfonylurea  Meglitinide  Biguanide  Thiazolidinedione  Dipeptidyl peptidase 4 (DPP4) inhibitor  Glucagon-like peptide-1 (GLP1) receptor agonist  Sodium-glucose transport protein 2 (SGLT2) inhibitor  Alpha glucosidase inhibitor, bile acid sequestrant |
| Long-acting Insulin Classes | Glargine (Lantus)  Basaglar  Toujeo  Degludec (Tresiba)  Detemir (Levemir) |
| Short/Rapid-acting Insulin Classes | Lispro (Humalog)  Aspart (Novolog)  Fiasp  Glulisine (Apidra)  Admelog |
| Fixed Dose Insulin Classes | Humulin 70/30  Lispro 50/50  Lispro 75/25  Aspart 70/30  NPH  Regular |
| CSII | ICD9 V45.85, V65.46  ICD10 Z96.41, Z46.81  Insulin pump  Medtronic  Animas  Tandem  Omnipod |

| **Supplemental Table 3 - Enrollment by Year** |  |  |  |
| --- | --- | --- | --- |
| **Year** | **Patients Entered**  **(N, %)** | **CSII Use**  **(N, %)** | |
| 2008 | 98 (12.2) | 11 (11.2) |  |
| 2009 | 108 (13.4) | 16 (14.8) |  |
| 2010 | 123 (15.3) | 20 (16.3) |  |
| 2011 | 108 (13.4) | 13 (12.0) |  |
| 2012 | 93 (11.6) | 8 (8.6) |  |
| 2013 | 92 (11.4) | 10 (10.9) |  |
| 2014 | 82 (10.2) | 14 (17.1) |  |
| 2015 | 58 (7.2) | 9 (15.5) |  |
| 2016 | 43 (5.3) | 4 (9.3) |  |
| **Total** | **805** | **105 (13.0)** |  |
